# High altitude pulmonary edema (HAPE) in a Himalayan trekker: a case report

**DOI:** 10.1186/2046-7648-3-6

**Published:** 2014-03-17

**Authors:** Promish Shrestha, Matiram Pun, Buddha Basnyat

**Affiliations:** 1 Nepal International Clinic, Travel and Mountain Medicine, Kathmandu, Nepal; 2 Mountain Medicine Society of Nepal (MMSN), Kathmandu, Nepal; 3 Mountain Medicine and High Altitude Physiology, University of Calgary, Calgary, Canada; 4 Oxford University Clinical Research Unit–Nepal (OCRU-Nepal), Patan Academy of Health Science (PAHS), Oxford, USA

**Keywords:** Acclimatization, Acute mountain sickness, Himalaya, Altitude sickness, Mountains Nepal, Pulmonary edema, Oxygen, Acetazolamide

## Abstract

**Introduction:**

High altitude pulmonary edema is a non-cardiogenic form of pulmonary edema that develops in unacclimatized individuals at altitudes over 2500 m. Early recognition of symptoms and immediate descent are important for successful treatment. Despite early signs and symptoms of high altitude illness, many trekkers tend to push themselves to the maximum limit. Some of them, such as the case reported here, choose to ascend on horse-back which is extremely dangerous and can be fatal.

**Case presentation:**

A 55 years of age Indian ethnic South African lady was emergency air-lifted from 4410 m altitude in the Nepal Himalayas to Kathamandu (1300 m) with a suspected case of high altitude pulmonary edema. She had continued ascending despite experiencing mild altitude symptoms at Namche (3440 m), and these symptoms worsened considerably at Tengboche (3860 m). At the very start of her trek, just after Lukla (2800 m), she suffered from sore throat, and had consequently begun a course of antibiotics (azithromycin) for a suspected throat infection. She had planned to continue ascending on horse back to complete the trek, however her condition deteriorated further and she had to be medically evacuated.

On admission to the clinic her axillary temperature was 99.4 F, blood pressure 120/60 mmHg, pulse rate 72/min, respiratory rate of 25 breaths/min, and pulse oximeter showed saturation of 90% on room air at rest. Right sided crackles on the axillary and posterior region were heard on chest auscultation. Heel to toe test showed no signs of ataxia. The chest radiograph showed patchy infiltrates on the right side. An echocardiogram was done which revealed a high pulmonary artery pressure of 50 mm of Hg. She was diagnosed as resolving high altitude pulmornay edema. She was treated with bed rest, supplemental oxygen and sustained release nifedipine 20 mg (orally) twice a day. On the third day her crackles had cleared significantly and repeat chest radiograph as shown showed remarkable improvement. She felt much better. A repeat echocardiogram revealed a normal pulmonary artery pressure.

**Conclusion:**

The case report highlights numerous points:

1) Many high altitude trekkers have invested significant time, money and physical efforts in in their ventures and are determined to ascend despite early warning and illnesses. 2) Despite no history of altitude illnesses in previous altitude exposure,inter-current illness (in this case a nonspecific respiratory tract infection) may contribute to the development of high altitude pulmonary edema. 3) Continuing ascent using other transport means, whilst suffering from symptoms of high altitude illness, worsens the condition and could be life threatening. 4) Acetazolamide does not prevent high altitude pulmonary edema–perhaps more so in the cases that have inter-current illness. 5) Descent is the golden rule in all altitude illnesses. Actually ‘descent’ is advised in any undiagnosed illness at high altitude among sojourners. 6) Finally, an experienced guide who has mountain medicine training is essential. They can be crucial in noticing early signs and symptoms of altitude illnesses to inform the client’s safety as in this case.

## Key messages of the case report

1) *Continuing ascent using various transport means (e.g. horse in this case) with high altitude illness symptoms worsens condition and could be life threatening*.

2) *Many high altitude trekkers have invested significant time, money and physical efforts in in their ventures and are determined to ascend despite early warning and illnesses*.

3) *Despite no history of altitude illnesses in previous altitude exposure,inter-current illness (in this case a nonspecific respiratory tract infection) may contribute to the development of high altitude pulmonary edema*.

4) *Acetazolamide does not prevent high altitude pulmonary edema–perhaps more so in the cases that have inter-current illness*.

5) *Descent is the golden rule in all altitude illnesses. Actually ‘descent’ is advised in any undiagnosed illness at high altitude among sojourners*.

6) *Finally, an experienced guide who has mountain medicine training is essential. They can be crucial in noticing early signs and symptoms of altitude illnesses to inform the client’s safety as in this case*.

## Background

High altitude pulmonary edema (HAPE) is a life threatening form of altitude illness [1,2]. Hypoxia induces uneven pulmonary vasoconstriction leading to extravasation of fluid from the pulmonary capillary beds leads to HAPE in an unacclamitized individual [3]. It may occur without the features of acute mountain sickness (AMS) such as headache, tiredness, nausea and dizziness [1,2]. It is important to recognize its early signs and symptoms as ascending with such symptoms can quickly turn a relatively mild altitude illness to severe pulmonary or cerebral edema. A concurrent respiratory tract infection (RTI) is an additional risk factor for HAPE [3].

Here we describe the development of HAPE in a 55 year old woman who was trekking in the Himalayas.

## Case presentation

A 55 years of age Indian ethnic South African lady presented in a travel medicine clinic in Kathmandu for her pre-hiking consultation. She was in good physical condition and had been on high-altitude trips before up to a maximum of 4600 m in Machu Pichu (Peru) without suffering from any symptoms of altitude illness.

This time she was trekking in the Khumbu region of Nepal with a group of fellow South Africans. She started her trek from an altitude of about 2800 m at Lukla and was doing well except for a sore throat which had started just after Lukla. She had begun a course of antibiotics (azithromycin) for this suspected throat infection. At Namche Bazaar (3440 m) where she stayed for 2 nights, she started to experience mild headaches, weakness, cough and nasal congestion but continued to go up to Tengboche (3860 m). At Tengboche, she experienced shortness of breath, bouts of coughing with sputum and the fatigue worsened. However after a night’s stay at Tengboche she continued to ascend to Dingboche (4410 m) where she had extreme fatigue, vomiting (once en route), coughing and difficulty breathing. She had no fever. Most of these symptoms were mild at rest but worsened upon the slightest exertion. She had started taking acetazolamide 250 mg twice a day and continued her azithromycin from Namche Bazaar. After the second night at Dingboche the patient was feeling worse with what she described as gurgling sounds arising from her chest and yet she was determined to continue to ascend on horseback the following day with the rest of the group. However, her discerning guide noted the seriousness of the condition and radioed his company in Kathmandu to arrange for a helicopter evacuation. She was transported by helicopter from Dingboche to Kathmandu (1300 m) and presented at the travel clinic. She felt improved after the descent.

**Figure 1:**
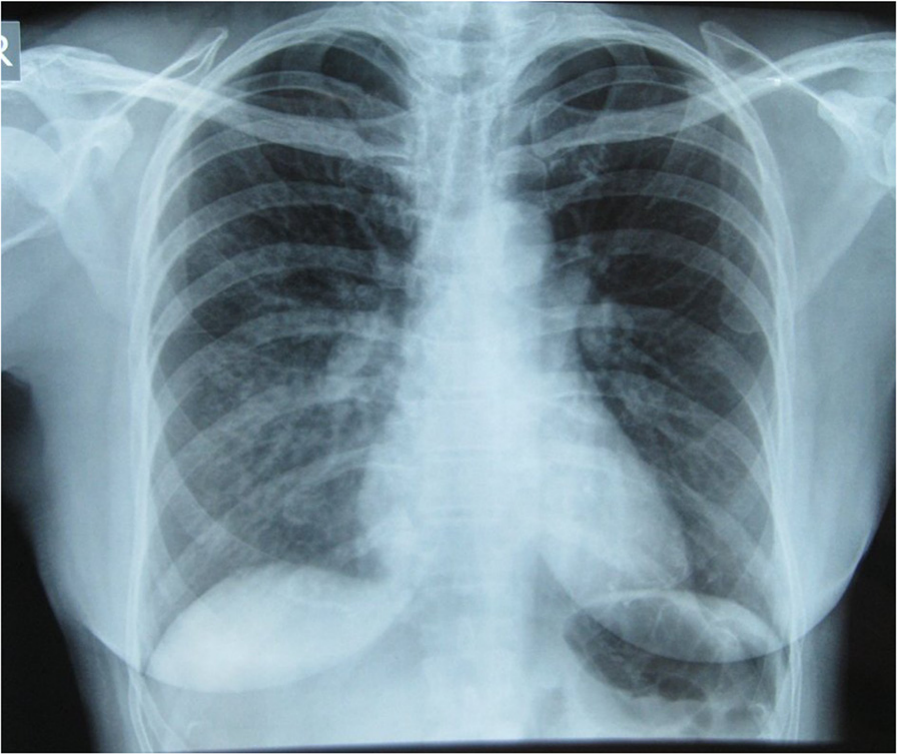
Initial Chest x-ray showing pulmonary infiltrates in the right lung especially in the right mid and lower lung zones indicative of pulmonary edema.

**Figure 2:**
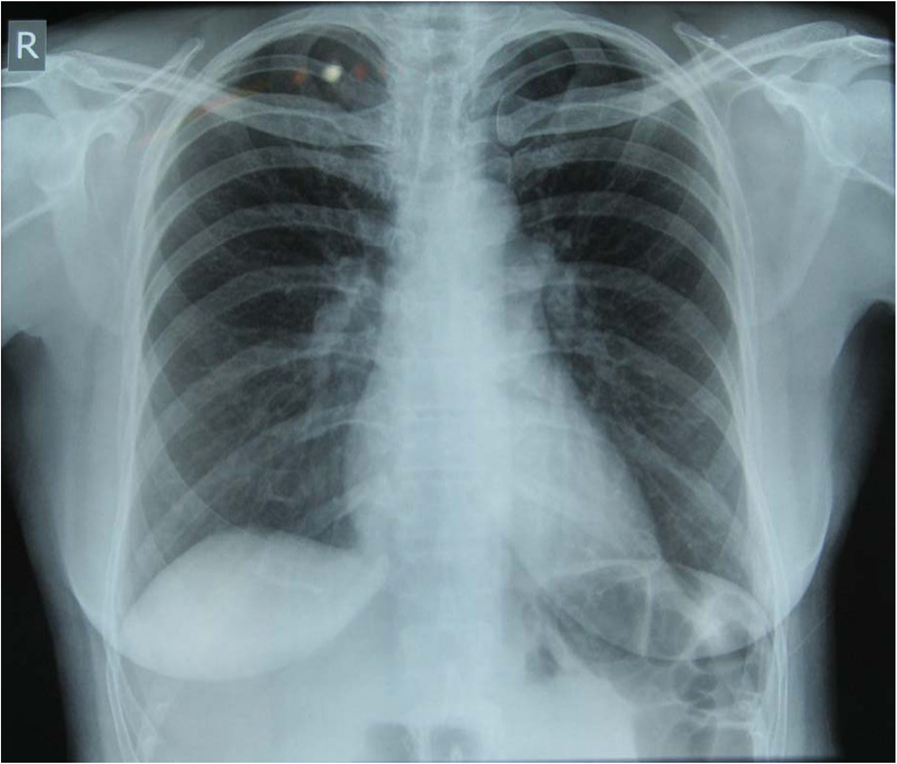
Repeat chest x- ray after 2 days showing rapid resolution of the edema.

On admission to the clinic her axillary temperature was 99.4 F, blood pressure 120/60 mmHg, pulse rate 72/min, respiratory rate of 25 breaths/min, and pulse oximeter showed saturation of 90% on room air at rest. Right sided crackles on the axillary and posterior region were heard on chest auscultation. Heel to toe test showed no signs of ataxia. The chest radiograph showed patchy infiltrates on the right side (Figure 1). An echocardiogram was done which revealed a high pulmonary artery pressure of 50 mm of Hg. She was diagnosed as resolving high altitude pulmornay edema (HAPE). She was treated with bed rest, supplemental oxygen and sustained release nifedipine 20 mg (orally) twice a day. On the third day her crackles had cleared significantly and repeat chest radiograph as shown showed remarkable improvement (Figure 2). She felt much better. A repeat echocardiogram revealed a normal pulmonary artery pressure.

## Discussion

This 55 year old woman continued to ascend despite early symptoms of HAPE. Pushing herself to further altitude on horseback (as planned) could have been fatal.

HAPE is a life-threatening non-cardiogenic pulmonary edema that often develops in non-acclimatized individuals going to high altitude. The prevalence of HAPE varies depending upon the setting. The incidence of HAPE among Himalayan trekkers and climbers in the Alps is around 4% [4] depending on the rate of ascent. It typically occurs in the first 2–4 days after arrival at altitudes above 2500 m [1,2]. A decrease in atmospheric partial pressure of oxygen causes diminished arterial oxygen saturation which further decreases during sleep, exercise and respiratory tract infections [5,6]. HAPE results after uneven hypoxic pulmonary vasoconstriction and increased pulmonary vascular pressure causes alveolar flooding with edema fluid [3]. Early signs and symptoms of mountain sickness if recognized early can prevent the development of severe forms when ascent is halted or descent is made.

The patient described here had symptoms of cough, headache, weakness and fatigue at an altitude of about 3400 m but assumed her symptoms to be due to non-specific RTI (that started at the very beginning of her trek in the Khumbu region at Lukla, 2800 m) and ascended further using antibiotics (azithromycin for the RTI) and acetazolamide for the prevention of acute mountain sickness (AMS). Unfortunately her symptoms worsened with shortness of breath at minimal exertion, extreme fatigue and persistent cough-features consistent with HAPE. Once altitude symptoms develop, they may quickly progress into serious conditions such as AMS, high altitude cerebral edema (HACE) and HAPE, if appropriate measures are not taken-stop ascending, pharmacological treatment and descent. Hence, it is important to emphasize that a further gain of altitude is always detrimental with altitude illness symptoms even while using acetazolamide. Ascending further using alternative transport means, as she had planned, can be catastrophic. Further, continued ascent would have increased her hypoxemia, enhanced her pulmonary artery pressures and worsened HAPE [3]. With further hypoxemia the subject could have developed a more complex syndrome of altitude illnesses (AMS, HAPE, and HACE) which could be fatal. It was correct judgment on the part of her guide to descend immediately in a helicopter.

Another factor that could have predisposed her to HAPE is a concurrent respiratory tract infection. A high rate of preceding RTI was found in children who developed HAPE [7]. Hence, this early inter-current illness nonspecific RTI might have contributed to the development of HAPE. Although HAPE is believed to be non-inflammatory in nature, but rather related to stress failure [8], a pre-existing inflammation may have caused the subject to be more susceptible to the stress failure. Acetazolamide used here might have prevented AMS and improved sleep quality [9,10] but does not seem to have prevented HAPE. Acetazolamide is useful in preventing and treating AMS but has no known role in HAPE [11]. Therefore, it becomes more important in such patients to make a descent once the symptoms of HAPE are recognized.

A prior history of HAPE is a significant predisposing factor rendering an individual HAPE susceptible [3]. However as in this case, people who have not developed HAPE in previous exposures to high altitude, may develop HAPE if factors favouring increased capillary permeability are present such as infection [3].

It is important to recognize HAPE and not confuse it with pneumonia. She was treated with oxygen and nifedipine after arrival to the travel clinic. Her follow up chest x-ray resolved completely after 2 days (Figure 1 and Figure 2). Chest x-ray changes of HAPE, unlike pneumonic consolidations, usually resolve in days with descent, and oxygen is the main treatment [12]. If they have no superadded infection, they do not require additional antibiotics [11,12]. In addition her high pulmonary artery pressure is very suggestive of HAPE and not a consolidation. Both the prompt resolution of the opacity on the chest X-ray and the initial increased pulmonary artery pressure are evidence of HAPE.

Other predisposing factors for HAPE include preexisting conditions or anatomical abnormalities. Both of these may cause increased pulmonary blood flow and/or intravascular pressures, even at altitudes less than 2500 m [13]. One well-studiedexample of a condition leading to HAPE is patent foramen ovale (PFO). The presence of PFO has been found to be four times higher in HAPE-susceptible individuals [3], and it is advisable that individuals with HAPE have an echocardiography to rule out any anatomical abnormalities of the heart.

## Conclusions

Many individuals have invested significant time, money and physical efforts to go high altitude. They will be determined to ascend even at the cost of one’s health. Continuing ascent in the face of worsening symptom by using other means of transport should always be discouraged. Although acetazolamide is used for prophylaxis and treatment of acute mountain sickness (AMS), it has no established role in the prevention and treatment of HAPE. Descent is the golden rule in all high altitude illnesses. It will be wise to descend for any undiagnosed illnesses at high altitude among sojourners. Oxygen, needs to be administered, nifedipine can be used as an adjunct, but descent remains an utmost priority. Hyperbaric bags e.g. Gamow Bag or Portable Altitude Chamber are useful (especially in cases of HAPE and HACE) to buy time for the rescue arrangements to wait.

## Consent

Written informed consent was obtained from the patient for publication of this manuscript and any accompanying images. A copy of the written consent is available for review by the Editor-in-Chief of this journal.

## Abbreviations

AMS: Acute mountain sickness; HACE: High altitude cerebral edema; HAPE: High altitude pulmonary edema; PFO: Patent foramen ovale; RTI: Respiratory tract infection.

## Competing interests

The authors declare that they have no competing interests.

## Authors’ contributions

PS and BB prepared the text and collected all the medical data. PS, BB and MP reviewed the literature, provided suitable references and assisted with the draft version of the paper. BB, PS, and MP reviewed and interpreted the X-Ray images and prepared them for the manuscript. BB and MP reviewed the paper and revised it to the final format. All authors read and approved the final manuscript.
